# Comparative Mitogenome Analyses of Subgenera and Species Groups in *Epeorus* (Ephemeroptera: Heptageniidae)

**DOI:** 10.3390/insects13070599

**Published:** 2022-06-30

**Authors:** Zhenxing Ma, Ran Li, Binqing Zhu, Xuhongyi Zheng, Changfa Zhou

**Affiliations:** 1 The Key Laboratory of Jiangsu Biodiversity and Biotechnology, College of Life Sciences, Nanjing Normal University, Nanjing 210023, China; a952718141@outlook.com (Z.M.); li471329014@163.com (R.L.); zxhy2000@outlook.com (X.Z.); 2 School of Life Sciences, Qufu Normal University, Qufu 273165, China; 3 Nanjing Institute of Environmental Sciences, Ministry of Ecology and Environment/State Environmental Protection Scientific Observation, Nanjing 210042, China; 18260029696@163.com

**Keywords:** gene rearrangement, intergenic spacer, phylogenetic relationship, mayfly, China

## Abstract

**Simple Summary:**

As one of the most species-rich genera of Ephemeroptera, *Epeorus* Eaton, 1881, was found to be widely distributed in Holarctic and Oriental regions, and nine subgenera have been reported. Previous phylogenetic studies of *Epeorus* were mainly focused on morphological characters or several gene fragments. Here, 15 mitogenomes of *Epeorus* are sequenced and the comparative mitogenome analysis of six subgenera is performed. The gene rearrangement of *trnI*-*trnM*-*trnQ*-NCR-*ND2* was first found in the genus. In addition, differences in genetic composition and codon usage between the species with this kind of rearrangement and other *Epeorus* species were observed. Phylogenetic analyses show that three subgenera, *Proepeorus*, *Belovius* and *Iron*, are not monophyletic groups, and our results imply that gill structures are not always appropriate for the classification of subgenera in *Epeorus*.

**Abstract:**

*Epeorus* Eaton, 1881 is a diverse mayfly genus in Heptageniidae comprising more than 100 species which are further divided into nine subgenera and several species groups. However, the classification and the phylogenetic relationships among them are still uncertain. Here, 15 complete mitochondrial genomes of *Epeorus* were sequenced and compared together with six available ones of same genus in the NCBI database. Based on morphological classification, the 21 mitogenomes were classified into six subgenera (*Proepeorus*, *Epeorus* s.str., *Belovius*, *Iron*, *Caucasiron* and *Siniron*) and four species groups (G1, G2, *montanus* and *longimanus*). Among all analyzed mitogenomes, the gene rearrangement of *trnI*-*trnM*-*trnQ*-NCR-*ND2* was first found occurring in three species of group G1, whereas the gene block *trnI*-*trnM*-*trnQ*-*trnM*-*ND2* was observed in all other mitogenomes of *Epeorus*. Furthermore, the genetic composition and codon usage of species in group G1 were also significantly different from all other *Epeorus* species, except group *longimanus*. The intergenic spacer between *trnA* and *trnR*, which has the stem-loop secondary structure, occurred in all 21 mitogenomes, and the sequences of stems and loops were conserved within species groups. Furthermore, the phylogenetic analyses strongly support the monophyly of all species groups, although three of six recognized subgenera *Proepeorus*, *Belovius*, and *Iron*, were shown as the non-monophyletic groups.

## 1. Introduction

Heptageniidae is known as one of the most species-rich families of mayfly (Ephemeroptera). They have dorsoventrally flattened nymphal bodies and are adapted to the aquatic habitats with swift currents [1]. Within this family, three subfamilies were taxonomically recognized: Ecdyonurinae, Heptageniinae and Rhithrogeninae [2,3]. *Epeorus* Eaton, 1881, a genus of the latter subfamily, has more than 100 described species all over the world, and most of them are distributed in Holarctic and Oriental regions [4,5,6,7,8]. Currently, nine subgenera and several species groups have been proposed for this genus [7,8,9,10,11]. Morphologically, the species of *Epeorus* can be divided into two types based on nymphal stage [9,10]: (1) Iron/g1 (see Kluge [10]), including subgenera *Iron*, *Ironopsis*, *Caucasiron*, *Alpiron* and *Siniron*, has a “suction disc” structure formed by gill lamellae; (2) all other species containing the subgenera *Epeorus* s.str., *Proepeorus*, *Belovius* and *Albertiron* are without the “suction disc”. Although the phylogenetic relationships within *Epeorus* have been investigated using morphological characters or a few gene fragments in some studies [9,10,11], the monophyly of those subgenera and species groups as well as their phylogeny are still controversial.

In recent years, more species and intermediate types of genus *Epeorus* have been reported, especially for the subgenus *Proepeorus*, a taxon characterized by plesiomorphies only [8]. Among Chinese *Proepeorus* species, two distinct groups were proposed, since they are significantly different from the type species: the (1) “single-spine” group (nymphs with single spines on abdominal terga, defined as species group “G1” in this work), and the (2) “paired-spines” group (nymphs have paired spines on abdominal terga, defined as species group “G2” here). The former group is similar to group *longimanus* (a species group of subgenus *Iron* [12]) morphologically, whereas the latter one resembles the subgenus *Siniron*, which also has “paired-spines”. Thus, more evidence is needed to confirm the definition of these subgenera and clarify their phylogenetic relationships. 

The mitochondrial genome is an important molecular marker that has been widely used to study the phylogenetic relationship of insects and other animal groups [13,14,15]. It contains 37 genes: 13 protein-coding genes (PCGs), 2 ribosomal RNA genes (rRNAs), 22 transfer RNA genes (tRNAs), and the A+T-rich region (or called control region, CR) [16,17]. The gene rearrangements and long non-coding regions (NCR) (or intergenic spacer, IGS) could also bring important information to the phylogenetic analyses [18,19,20]. The studies on the mitochondrial genomes of Ephemeroptera and Heptageniidae have increased in recent years [18,19,21,22,23]. The duplication of *trnM* has been observed in all reported mitochondrial genomes of Rhithrogeninae and Ecdyonurinae species (except *Paegniodes cupulatus*), and the genes were arranged as CR*-trnI-trnM-trnQ-trnM-ND2*, and another rearrangement (CR*-trnI-trnM-trnQ-*NCR*-ND2*) was reported in the Heptageniinae species [18,19,24,25,26]. In addition, a conserved intergenic spacer (IGS) between *trnA* and *trnR* was also observed in all heptageniid species [18,19]. For *Epeorus*, there are currently 16 published mitochondrial genomes, of which only seven species have been identified in GenBank. Hence, the phylogenetic relationship and comparison between the subgenera and species groups within *Epeorus* based on mitogenome data are still poorly understood.

Here, 21 complete mitogenome sequences belonging to six subgenera of *Epeorus* were compared and analyzed, including six previously reported ones and 15 newly obtained sequences in this work. The mitogenomic features of different subgenera or species groups within *Epeorus* were compared and their phylogenetic relationships were also reconstructed. The results can not only expand our knowledge on the mitogenomic features of Heptageniidae and *Epeorus*, but also provide new information for the definition of the subgenus within *Epeorus*.

## 2. Materials and Methods

### 2.1. Sample Collection, Identification and DNA Extraction

All specimens were collected in China between 2014–2021 (see Table S1). The morphological identification was based on Kluge [10] and Ma et al. [7,8]. All samples were preserved in 100% ethanol and stored at −20 °C in the Mayfly Collection of Nanjing Normal University. The genomic DNA was extracted from the muscle tissue of each species using TIANamp Genomic DNA Kit (TIANGEN, Beijing, China). DNA concentrations were determined by Nanodrop 2000 spectrophotometer. 

### 2.2. Mitogenome Sequencing and Assembly

The genomic DNA of all species was sequenced using next-generation sequencing (NGS) on Illumina NovaSeq platform. TruSeqTM DNA Sample Prep Kit was used for library construction of each sample (insert size 400 bp). All libraries were then sequenced based on the PE150 mode (paired-end, 2 × 150 bp). More than 4GB raw data of each sample were trimmed so that adapter contamination, short and low-quality reads were removed using Fastp [27]. NOVOPlasty 4.2.1 was used for the de novo assembly of mitogenome [28], with the parameter k-mer = 33 and the *COI* gene of *Epeorus dayongensis* (accession number: MT112895) as the seed sequence. In order to verify the accuracy of NGS sequencing results, three gene fragments of all 15 samples were sequenced using Sanger sequencing: fragment of *COI*, fragment of *rrnL*, and fragment containing block (*trnI-trnM-trnQ*-*trnM*/NCR-*ND2*). Two pairs of general primers were used for the first two fragments, and fifteen pairs of specific primers were designed for the last one (shown in Table S5) [29,30]. r-Taq polymerase (Takara, Beijing, China) was used for the polymerase chain reaction (PCR) based on the method of Yanai et al. [31].

### 2.3. Mitogenome Annotation and Analyses

The mitogenomes were annotated using MITOS WebServer initially [32], and checked by alignments with available sequences of *Epeorus* from GenBank using ClustalW in MEGA11 [33]. The secondary structures of tRNAs were determined using tRNAscan-SE 2.0 and MITOS WebServer [34]. The circular maps of mitogenomes were portrayed using the visualize module of MitoZ [35].

Nucleotide composition and relative synonymous codon usage (RSCU) were calculated using MEGA11 [33]. The AT-skew and GC-skew were calculated by the formulas: AT-skew = (A − T)/(A + T) and GC-skew = (G − C)/(G + C) [36]. A sliding window analysis of 200 bp (step size 20 bp) was used to estimate nucleotide diversity (Pi) of 13PCGs by DnaSP V6 [37]. Genetic distances of the mitogenomes of *Epeorus* species were estimated using MEGA11 based on Kimura 2-parameter model [38]. The ratio of non-synonymous/synonymous rate (ratio of Ka/Ks) of 13 PCGs was conducted by DnaSP V6.

### 2.4. Phylogenetic Analysis

A total of 24 mitogenomes of Heptageniidae were used for the phylogenetic reconstruction, including two species of Ecdyonurinae and one species of Rhithrogeninae as out groups. A total of 15 newly sequenced mitogenomes and 6 available mitogenomes of *Epeorus* were selected as the ingroup taxa. All sequences of *Epeorus* used in this work have been morphologically identified, and their subgenera have been confirmed. We also found that *Epeorus bifurcatus* (GenBank accession number: MW381293) reported previously should be identified as *Epeorus falcatus* according to the morphological characters [8], and it has been modified in this work (Table 1). Some other available mitochondrial genomes of *Epeorus* were not used in this work because we cannot guarantee the accuracy of their morphological identification.

The nucleotide sequences of 13 PCGs were aligned with MAFFT [39] L-INS-i (accurate) strategy and codon alignment mode in PhyloSuite v1.2.2 [40]. Sequences of rRNAs were aligned with MAFFT [39] G-INS-i (accurate) strategy. The ambiguously sites of all alignments were removed using Gblocks 0.91b [41]. Individual genes were than concatenated using PhyloSuite v1.2.2. Two datasets were used for the reconstruction of phylogenetic relationship: the (1) PCGs matrix, including all codon positions of 13 protein-coding genes; and the (2) PR matrix, containing 13 PCGs and two rRNAs. For the phylogenetic reconstruction, Bayesian Inference (BI) and Maximum Likelihood (ML) analyses were performed based on both datasets. The partition model of each dataset was selected by the PartitionFinder2 based on BIC (Bayesian information criterion) criterion and greedy algorithm (Tables S6 and S7) [42]. BI phylogenies were reconstructed using MrBayes 3.2.6 through online CIPRES Science gateway [43,44], with the following settings: two parallel runs with four Markov chains were run for 10 million generations (with sample frequency of 1000), with a burn-in of 25% trees. RAxML 8.2.0 were used for ML analyses, with the model of GTRGAMMAI and 1000 bootstrap replicates [45]. FigTree 1.4.2 (http://tree.bio.ed.ac.uk/software/figtree/; accessed on 5 July 2021) was used for the edit of phylogenetic trees.

## 3. Results and Discussion

### 3.1. Mitogenome Organization and Composition

**Figure 1:**
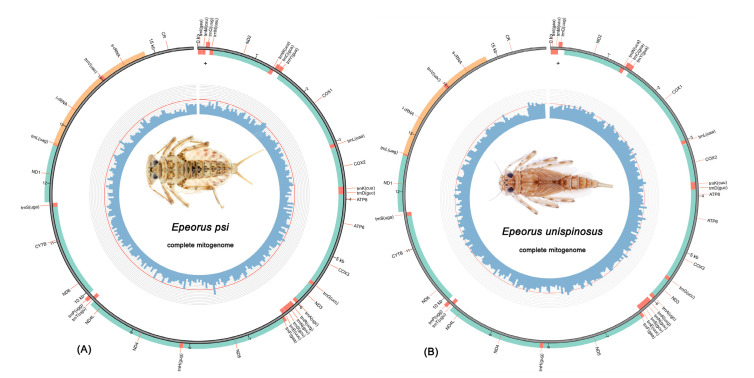
Mitochondrial maps for two types of rearrangement in *Epeorus*: (**A**) *E. psi* (with gene block *trnI-trnM-trnQ-trnM-ND2*); (**B**) *E. unispinosus* (with gene block *trnI-trnM-trnQ*-NCR-*ND2*).

All mitogenomes of 15 new samples were sequenced and compared with 6 available mitogenomes of *Epeorus*, and all sequences were deposited in GenBank (GenBank accession number: OK495692–OK495706). The total lengths of the 21 mitogenomes ranged from 15,338 bp (*Epeorus carinatus*) to 15,849 bp (*Epeorus unispinosus*), and the variation was mainly caused by the different sizes of CRs (Table S2). Three mitogenomes of species group G1 (*E. gibbus*, *E. unispinosus* and *E.* sp. 1) contained 37 genes (13 PCGs, 22 tRNAs and two rRNAs), whereas all other mitogenomes of *Epeorus* comprised an additional *trnM* (13 PCGs, 23 tRNAs and two rRNAs), which conformed to most Heptageniidae reported previously [18,19] (Figure 1). Among all mitogenomes, most genes (9 PCGs and 14 or 15 tRNAs) were encoded on the major strand, and the remaining genes were encoded on the minor strand.

**Figure 2:**
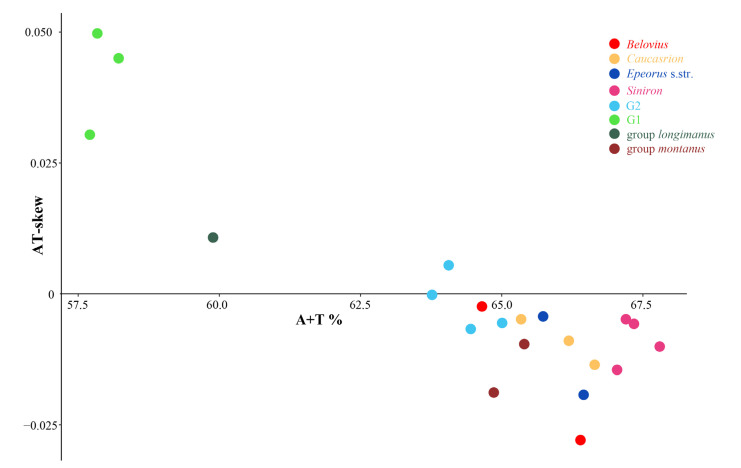
Scatterplots of A + T content and value of AT-skew for whole mitogenomes of *Epeorus*.

In addition to the genetic composition, the A + T content of group G1 was also significantly different from other *Epeorus* species (except *E. alexandri*, group *longimanus*). For most *Epeorus* species, the A + T content ranged from 63.8% (*E. rhithralis*, group G2) to 67.8% (*E. herklotsi*, *Siniron*); whereas in group G1 and *longimanus* (*E. alexandri*), the A + T content ranged from 57.7% (*E. unispinosus*) to 59.9 % (*E. alexandri*) (Figure 2). Similar variations of A + T content also occurred in PCGs and CR (Table S2). Additionally, most mitogenomes of *Epeorus* showed negative AT-skews (−0.028 to −0.0002), except for group G1 (0.03 to 0.045), *longimanus* (0.01), and *Epeorus aculeatus* (0.005). The GC-skews of all mitogenomes were obviously negative (−0.206 to −0.315) (Table S2). 

In summary, group G1 is different from other *Epeorus* species in terms of genetic composition, A + T content, and AT-skews. Among other species of *Epeorus*, the closest one to group G1 is *E. alexandri* (group *longimanus*), and this is also consistent with the morphological characters: species of group G1 possess similar mouthparts and genitalia to species of group *longimanus* [8].

### 3.2. Analysis of Protein-Coding Genes

**Figure 3:**
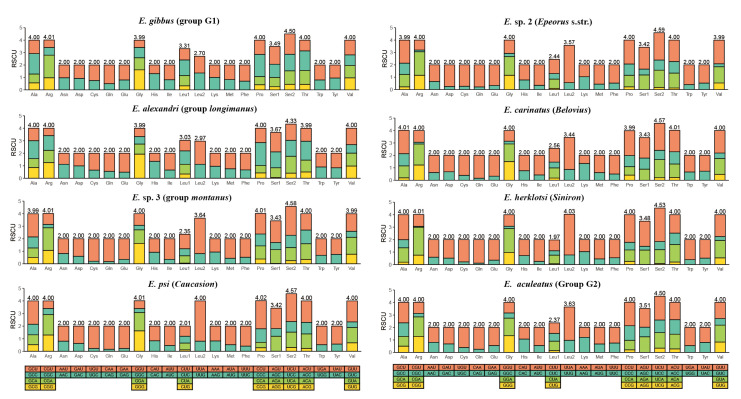
Relative synonymous codon usage (RSCU) of the mitogenomes of eight representative *Epeorus* species (including all species groups involved in this work).

The total length of PCGs for the majority of *Epeorus* mitogenomes was 11,217 bp, except for *E. gibbus* (11,215 bp), *E. unispinosus* (11,229 bp), and *E.* sp.1 (11,226 bp). Both the AT-skews and GC-skews of all samples were negative (Table S2), and this is consistent with most heptageniids in previous studies [18,19]. The start codons of most PCGs were conservative in *Epeorus* species (Table S3), containing ATG (*COII*, *COIII*, *CYTB*, *ND1*, *ND4*, *ND4L*), ATA (*ATP6*), and ATT (*COI*). For *ATP8*, *ND2*, and *ND5*, two types of start codons were found (ATG, GTG). The start codon ATC was only found in *ND3* of five species (*E. gibbus*, *E.* sp. 1, *E. alexandri*, *E. dayongensis*, and *E. tuberculatus*), whereas ATG was used as the start codon of *ND3* only in *Epeorus* s.str. (*E. melli* and *E.* sp. 2) and *E. carinatus*. TTG was used as the start codon only in *ND6* of *E. unispinosus*. Most PCGs stopped with TAA or TAG codons, and the incomplete terminating codon T was only found in *COII*, *ND4*, *ND5*, and *CYTB*, and it was consistent with the heptageniid mitogenomes sequenced previously [18,19]. The relative synonymous codon usage (RSCU) of 21 *Epeorus* mitogenomes was calculated and compared (Table S4, Figure 3). The RSCU values of several codons like CUC (Leu1), CCC (Pro), AGC (Ser1), AGG (Ser1), and GGC (Gly) in group G1 and *longimanus* were significantly greater than that of all other *Epeorus*, and this is consistent with the relatively higher C + G contents in PCGs of group G1 and *longimanus*. 

**Figure 4:**
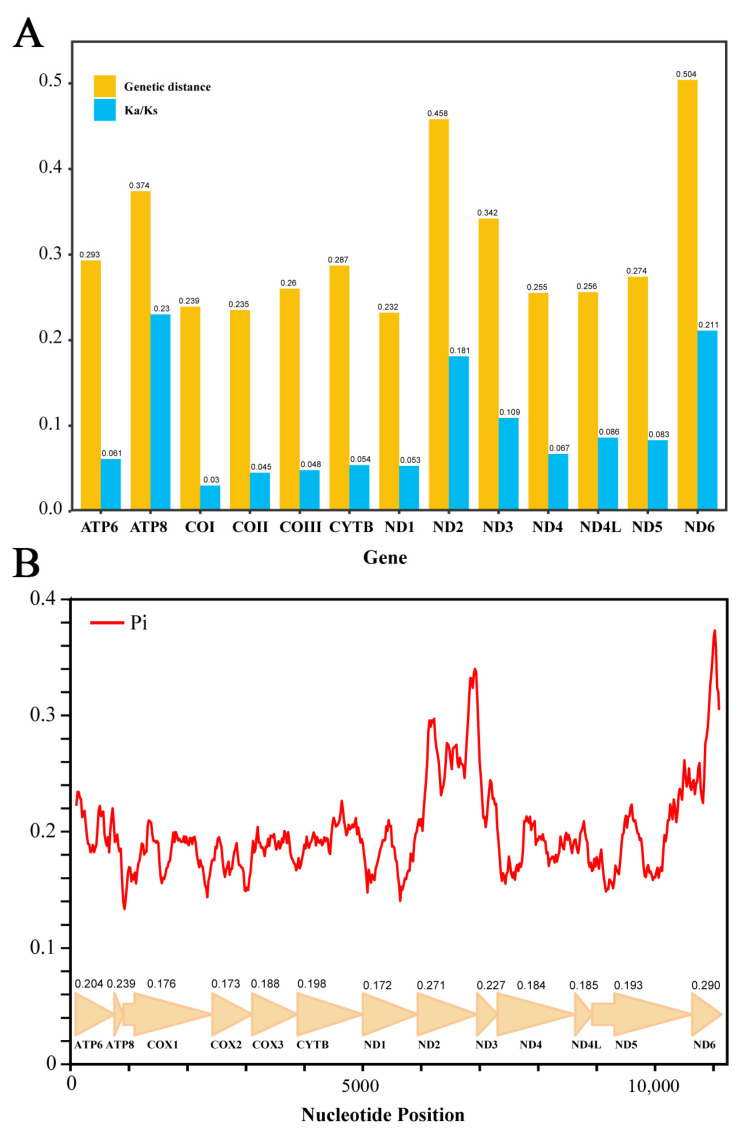
(**A**) Ratio of non-synonymous (Ka) to synonymous (Ks) substitution rates and average genetic distance of 13 PCGs among 21 *Epeorus* mitogenomes. (**B**) Sliding window analysis of 13 aligned PCGs among 21 *Epeorus* mitogenomes. The red line shows the value of nucleotide diversity (Pi).

The nucleotide diversity (Pi) of 13 aligned PCGs among all 21 mitogenomes was calculated by a sliding window analysis (Figure 4). In all PCGs, *ND6* (Pi = 0.29), *ND2* (Pi = 0.271), and *ATP8* (Pi = 0.239) were showing a relatively high nucleotide diversity, whereas the *ND1* (Pi = 0.172), *COI* (Pi = 0.176), and *COII* (Pi = 0.173) had comparatively low Pi value. Moreover, the pairwise genetic distances of these 13 PCGs also showed a similar result: *ND2*, *ND6*, and *ATP8* have high average values of distances, whereas *ND1*, *COII*, and *COI* showed relatively low distance values (Figure 4). Furthermore, the ratio of Ka/Ks was calculated to estimate the selective pressure of all 13 PCGs. Here, all the PCGs evolved under a purifying selection (Ka/Ks < 0.5) ranged from a strongly purifying selection (0.03 of *COI* and 0.045 of *COII*) to a relaxed purifying selection (0.23 of *ATP8*, 0.21 of *ND6*, and 0.18 of *ND2*) (Figure 4). The results of nucleotide diversity, genetic distance, and ratios of Ka/Ks of *Epeorus* showed similar tendency with the analyses of Heptageniidae [18]. As the relatively conserved genes with a slow evolution rate, *COI*, *COII*, and *ND1* could be selected as potential barcoding markers for species identification in *Epeorus*.

### 3.3. Transfer and Ribosomal RNA Genes

Except for the three species of group G1, all other *Epeorus* have an additional *trnM* besides 22 typical tRNAs genes. The length of tRNAs ranged from 63 to 71 bp, and all of them were folded into the typical clover-leaf secondary structure, except *trnS1*. As observed in other insects and Ephemeroptera, the DHU arm were also missing in *trnS1* of all *Epeorus* mitogenomes. Two rRNAs were encoded on the N-strand in all 21 mitogenomes. The *rrnL* was located between *trnL1* and *trnV*, with the sizes ranging from 1272 to 1284 bp. The *rrnS* was located between *trnV* and control region, and the sizes ranged from 779 to 787 bp. This indicates that the sizes of the *rrnL* of three species in *Siniron* (from 1272 to 1273 bp) were significantly smaller than that of all other *Epeorus* mitogenomes (from 1279 to 1284 bp), which might be a feature that can be used to identify the subgenus *Siniron*.

### 3.4. Non-Coding Regions

The non-coding regions of mitogenomes of *Epeorus* consist of control regions and several IGSs. The control region of *Epeorus* mitogenomes is located between *rrnS* and *trnI* genes, and is the longest non-coding region in the whole mitogenome. The total length of CR of 21 mitogenomes ranged from 504 (*E. carinatus*) to 1042 bp (*E. unispinosus*). Similar to the case of the A + T contents in total mitogenomes and PCGs, the A + T contents in CR of group G1 and *longimanus* (range from 58.9% to 67.8%) were also obviously smaller than other *Epeorus* mitogenomes (range from 72.8% to 78.9%) (Table S2). Moreover, compared to other genera in Heptageniidae [18,19], only the *Epeorus* species had a relatively high A + T contents in the control region, which is always <70% in other Heptageniidae species. 

**Figure 5:**
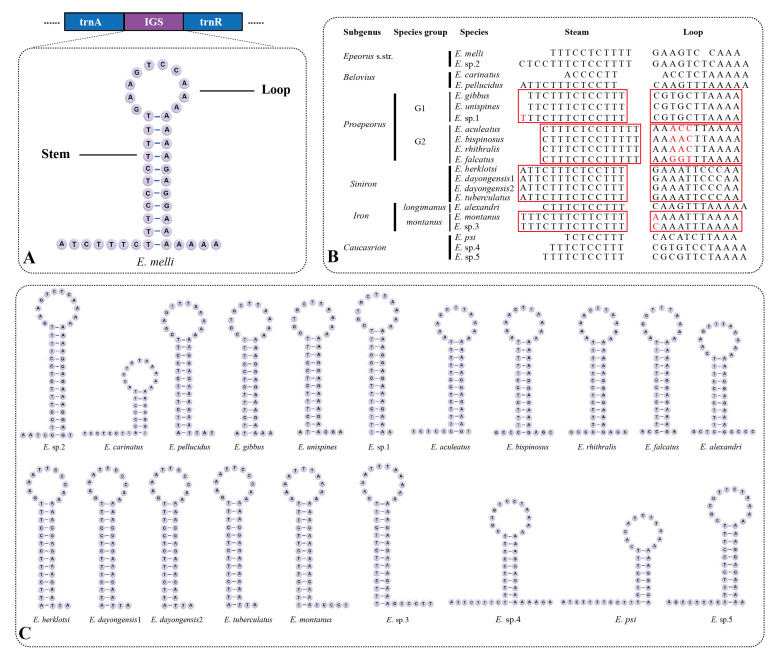
The intergenic spacers (IGS) between *trnA* and *trnR* in *Epeorus* mitogenomes: (**A**) The stem-loop structure; and the (**B**) alignment of the sequences for stems and loops among 21 *Epeorus* mitogenomes. The conserved sequences are in red boxes and the variable sites are shown in red. (**C**) The stem-loop structure of all IGS among 21 mitogenomes.

The long IGS between *trnA* and *trnR* is seen as the molecular synapomorphy of the family Heptageniidae [18,19], and it was also observed in all 21 *Epeorus* mitogenomes, with lengths ranging from 33 to 46 bp. They can be folded as stem-loop secondary structures (Figure 5). By comparing the IGSs of all 21 mitogenomes, we found that the sequences of stems and loops are variable between different species within a certain subgenus (except *Siniron*) but conserved within species groups or species (Figure 5B). It seems that the IGS between *trnA* and *trnR* may be a useful marker for the identification of species group (or species) in *Epeorus*. 

### 3.5. Gene Arrangement

According to the previous studies of heptageniid mitogenomes, only one species (*Paegniodes cupulatus*) had the gene arrangement that identifies with the ancestral gene order, and all other mitogenomes of Rhithrogeninae and Ecdyonurinae species have an extra *trnM* between *trnQ* and *ND2*, forming the gene cluster (CR-*trnI-trnM-trnQ-trnM-ND2*). In addition, another rearrangement (CR-*trnI-trnM-trnQ*-NCR-*ND2*) was only found in the species of Heptageniinae. In other words, until now, only two types of gene rearrangements have been observed in Heptageniidae [18,19]. 

In this work, most mitogenomes of *Epeorus* had the extra *trnM* and the gene block (CR-*trnI*-*trnM-trnQ*-*trnM*-ND2), which agreed with the previous research. Moreover, the other rearrangement was also first found in *Epeorus* (only for the three species of group G1): the *trnM* between *trnQ* and *ND2* was replaced by a non-coding region (range from 39 to 41 bp), forming the gene cluster (CR-*trnI-trnM-trnQ*-NCR-*ND2*), which previously only occurred in species of Heptageniinae. Similar to other species of Heptageniinae [19], the NCR between *trnQ* and *ND2* in groups G1 also showed a low similarity to other adjacent genes, and the sequences were not conserved among the three species in group G1. Therefore, we assume that the NCR between *trnQ* and *ND2* in group G1 was the sequence formed by the loss of *trnM*. It seems that the same gene cluster (CR-*trnI-trnM-trnQ*-NCR-*ND2*) in the mitogenomes of *Epeorus* and Heptageniinae were formed independently and should be regarded as the convergent evolution or homoplasy between these two taxa. A similar inversion in *trnI-trnQ-trnM* (ancestral order) for *trnI-trnM-trnQ* also occurs in 5 other different orders [46].

### 3.6. Phylogenetic Relationship

**Figure 6:**
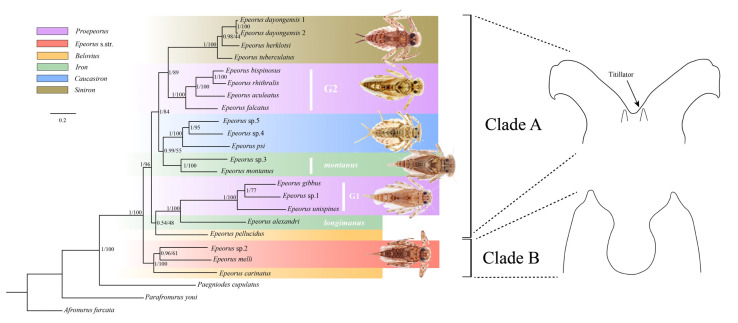
Phylogenetic relationships of *Epeorus* constructed by BI and ML methods based on datasets PCGs. Numbers separated by a slash on node are posterior probability and bootstrap values. The line drawings represent two types of genitalia of the clade A and B.

The two phylogenetic trees (BI and ML) based on dataset PCGs have the same topology as the ML tree of dataset PR (Figure 6). Moreover, the position of *E. pellucidus* in the BI tree of dataset PR is different from the former three analyses (Figure S1). The monophyly of genus *Epeorus* was highly supported in all analyses, and each tree was divided into two main clades: group G1 + group G2 + *Siniron* + group *montanus* + group *longimanus* + *Caucasiron* + *E. pellucidus* (clade A) and *Epeorus* s.str. + *E. carinatus* (clade B) (Figure 6). The monophyly of the two main clades were well supported in all trees but two species of *Belovius* (*E. carinatus* and *E. pellucidus*) were placed into different clades. In previous studies, the identification of *Belovius* was mainly based on the structure of the gills in the nymphal stage, whereas the characters of adults were often overlooked [10]. Here, we found that *E. carinatus* can be clustered with *Epeorus* s.str. based on the similar structure of the penis (without titillators, the imaginal characters of *E. carinatus* is according to unpublished data) in the adult stage, whereas all species of clade A (including *E. pellucidus*) possessed the titillators (Figure 6). Moreover, the two-clades pattern was also reflected in the start codon of *ND3*; ATG was only used as the start codon in clade B, whereas ATT and ATC were used as the start codons of all species in clade A (Table S3).

Within clade A, the position of *E. pellucidus* was unstable, in that, both topologies were not well supported in the node containing this species (Figure 6, Figures S1 and S2). Consequently, more samples of the *Belovius* species in further research were needed to confirm this place with confidence. The monophyly of all species groups in clade A were highly supported in all analyses (Figure 6, Figures S1 and S2). Group *longimanus* clustered with group G1 consistently, and this coincided with the morphological evidence proposed in Ma et al. [8]. In addition, these two groups were also very close in terms of the A + T content and codon usage bias, which were significantly different from all other *Epeorus* in this work. Moreover, group G2 was highly supported as the sister clade to the subgenus *Siniron*, and this also verified the hypothesis that the character of “paired spines” on abdominal terga in nymphs is the synapomorphy of these two lineages. Group *montanus* clustered with *Caucasiron* in all analyses but was only well supported in BI tree based on the PCG dataset, whereas the monophyly of *Siniron* + group G2 + group *montanus* + *Caucasiron* was well supported in all analyses. 

Except for *Caucasiron*, *Siniron* and *Epeorus* s.str., all other subgenera involved in this work were shown as non-monophyletic groups according to the phylogenetic trees reconstructed here (Figure 6). The result implies that gill structures or characters were not always appropriate for the classification of subgenera. A similar result was also obtained by Hrivniak et al. [11], who found that the “suction disc” of the gill structure is a homoplasy of several unrelated lineages according to the phylogenetic analysis based on five gene fragments. Thus, for the definition or classification of subgenera within *Epeorus*, a comprehensive consideration of more morphological features (e.g., mouthparts, abdominal spines and genitalia of adults) and molecular evidence is necessary.

## 4. Conclusions

In this study, 21 mayfly mitogenomes (including 15 newly sequenced) of the genus *Epeorus* were analyzed and compared. The rearrangement of *trnI-trnM-trnQ-*NCR*-ND2*, which was observed only in the species of subfamily Heptageniinae previously, was first found in *Epeorus* (Rhithrogeninae), but restricted to species group G1 (nymphal terga with single median spine). Furthermore, the genetic composition and codon usage bias of species in group G1 also displayed distinct differences compared with all other *Epeorus* species, except group *longimanus*. The close relationship between group G1 and *longimanus* was also strongly supported by the phylogenetic topologies. Moreover, the monophyly of all species groups and cluster of group G2 (nymph with a pair of abdominal spines on terga) + subgenus *Siniron* also had strong branch support. In contrast, three subgenera (*Proepeorus*, *Iron*, and *Belovius*) were found to be non-monophyletic. It seems that the traditional morphological features (such as the gill structure of nymphs) used in the classification of subgenera in *Epeorus* should be updated and molecular evidence should also be considered in defining a subgenus in future works. 

## Figures and Tables

**Table 1:** GenBank accession numbers of species used in this study.

Family	Genus	Subgenus	Species Group	Species	GenBank ID
Heptageniidae	*Epeorus*	*Proepeorus*	G1	*Epeorus gibbus*	OK495692 (this study)
			G1	*Epeorus unispinosus*	OK495693 (this study)
			G1	*Epeorus* sp. 1	OK495694 (this study)
			G2	*Epeorus aculeatus*	OK495695 (this study)
			G2	*Epeorus bispinosus*	OK495696 (this study)
			G2	*Epeorus rhithralis*	OK495697 (this study)
			G2	*Epeorus falcatus*	MW381293
		*Epeorus*		*Epeorus melli*	MW381294
				*Epeorus* sp. 2	OK495698 (this study)
		*Belovius*		*Epeorus carinatus*	MT112896
				*Epeorus pellucidus*	MW381296
		*Iron*	*longimanus*	*Epeorus alexandri*	OK495699 (this study)
			*montanus*	*Epeorus montanus*	MW381295
			*montanus*	*Epeorus* sp. 3	OK495700 (this study)
		*Siniron*		*Epeorus herklotsi*	OK495701 (this study)
				*Epeorus dayongensis* 01	MT112895
				*Epeorus dayongensis* 02	OK495703 (this study)
				*Epeorus tuberculatus*	OK495702 (this study)
		*Caucasiron*		*Epeorus psi*	OK495704 (this study)
				*Epeorus* sp. 4	OK495705 (this study)
				*Epeorus* sp. 5	OK495706 (this study)
Out group					
	*Paegniodes*			*Paegniodes cupulatus*	MW381300
	*Parafronurus*			*Parafronurus youi*	EU349015
	*Afronurus*			*Afronurus furcata*	MW381292

## Data Availability

The data that support the findings of this study are openly available in National Center for Biotechnology Information at https://www.ncbi.nlm.nih.gov/nuccore (accessed on 1 December 2021), reference numbers OK495692–OK495706.

## Supplementary Materials

The following supporting information can be downloaded at: https://www.mdpi.com/article/10.3390/insects13070599/s1, Figure S1: Phylogenetic relationships of *Epeorus* constructed by BI method based on datasets PR, node numbers represent the values of posterior probability; Figure S2: Phylogenetic relationships of *Epeorus* constructed by ML method based on datasets PR, node numbers represent the values of bootstrap; Table S1: Collection of information of Epeorus species in this study; Table S2: Nucleotide composition of 21 *Epeorus* mitogenomes; Table S3: Start and stop codons of 13 PCGs; Table S4: Codon count and relative synonymous codon usage (RSCU) of 21 *Epeorus* mitogenomes; Table S5: Primers for mitogenomes of *Epeorus* used in this study; Table S6: Partition schemes and best-fitting models selected in PCG dataset; Table S7: Partition schemes and best-fitting models selected in PR dataset.
