# Supplementary material for: Comparative Mitogenome Analyses of Subgenera and Species Groups in Epeorus (Ephemeroptera: Heptageniidae)
Source: Insects. 2022 Jun 30;13(7):599. doi: 10.3390/insects13070599 (PMC9317806; doi:10.3390/insects13070599)
Supplement: Supplementary file 1 [file Figures S1.pdf › Figures S1.pdf]

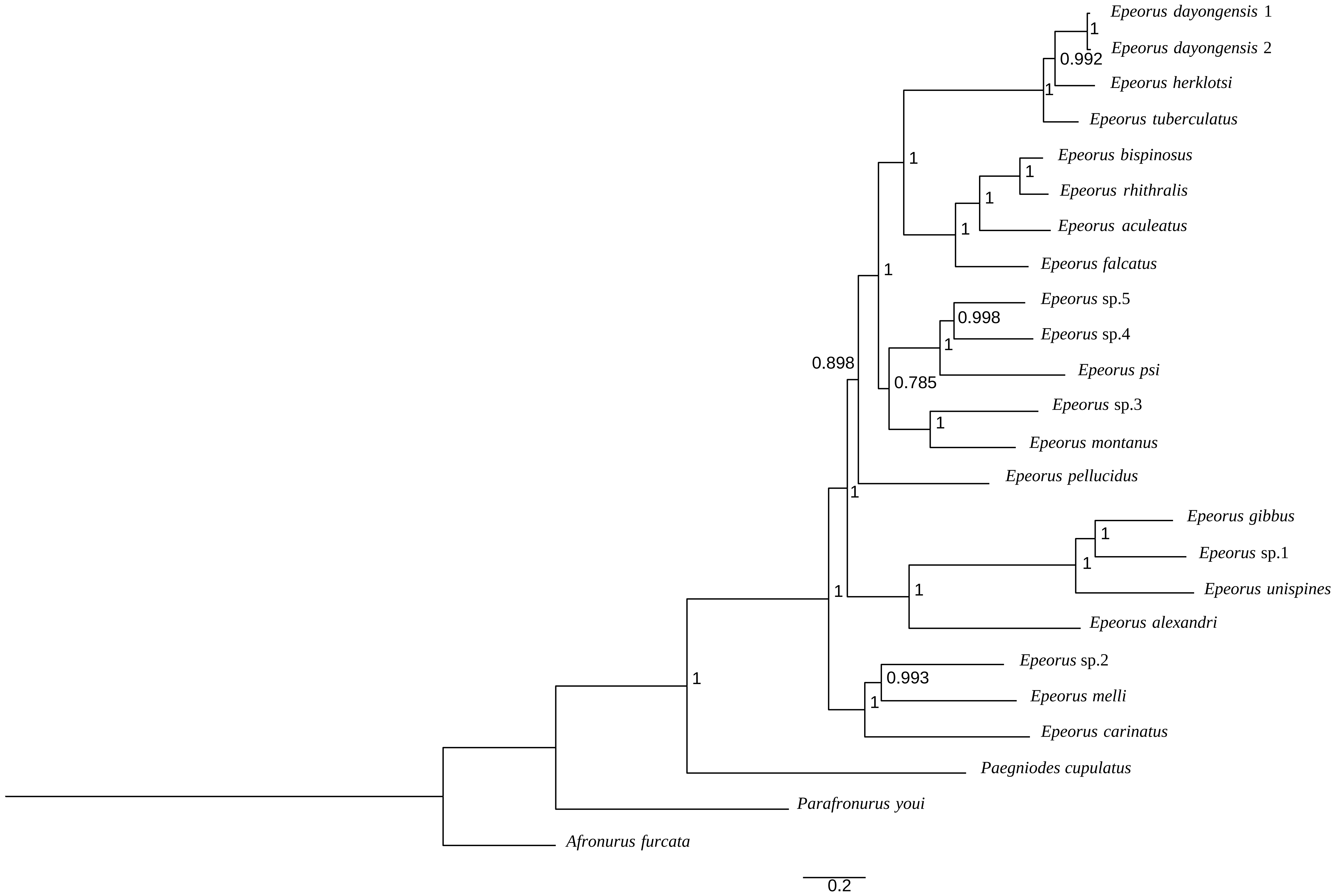


**Figure S1:** Phylogenetic relationships of *Epeorus* constructed by BI method based on datasets PR, node numbers represent the values of posterior probability;
