# Supplementary material for: Comparative Mitogenome Analyses of Subgenera and Species Groups in Epeorus (Ephemeroptera: Heptageniidae)
Source: Insects. 2022 Jun 30;13(7):599. doi: 10.3390/insects13070599 (PMC9317806; doi:10.3390/insects13070599)
Supplement: Supplementary file 1 [file insects-13-00599-s001.zip › Figures S2.pdf]

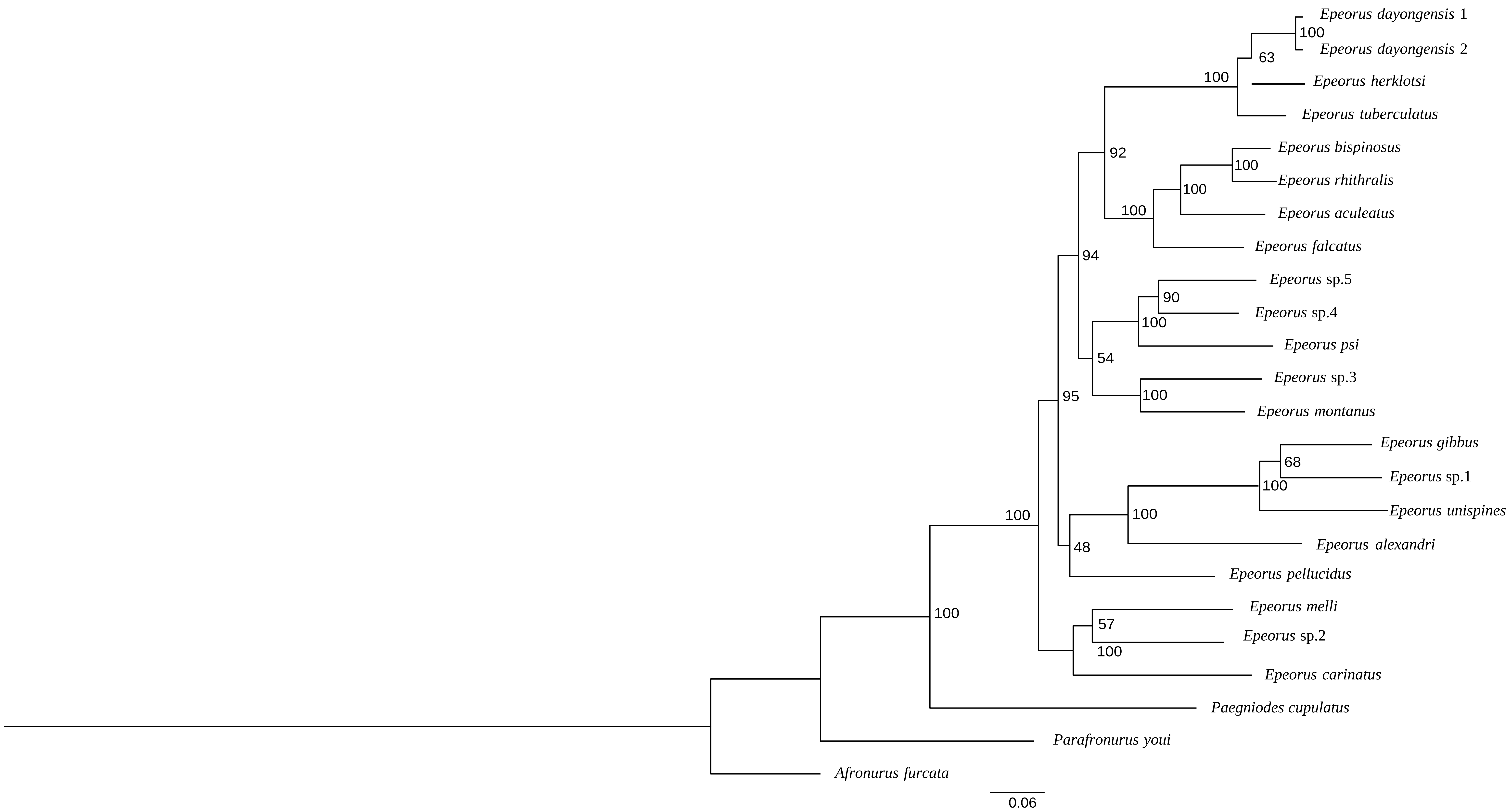

Figure S2: Phylogenetic relationships of *Epeorus* constructed by ML method based on datasets PR, node numbers represent the values of bootstrap;
