# Supplementary material for: Comparative Mitogenome Analyses of Subgenera and Species Groups in Epeorus (Ephemeroptera: Heptageniidae)
Source: Insects. 2022 Jun 30;13(7):599. doi: 10.3390/insects13070599 (PMC9317806; doi:10.3390/insects13070599)
Supplement: Supplementary file 1 [file insects-13-00599-s001.zip › Table S1.pdf]

Table S1 Collection information of *Epeorus* species in this study

No.	Species	Country	Province	Location	Date
1	*Epeorus gibbus*	China	Zhejiang	Hangzhou City, Tianmu Mountain	2019-IV-5
2	*Epeorus unispinosus*	China	Zhejiang	Hangzhou City, Tianmu Mountain	2021-IV-5
3	*Epeorus* sp.1	China	Zhejiang	Hangzhou City, Tianmu Mountain	2020-IV-4
4	*Epeorus aculeatus*	China	Yunnan	Pingbian County	2020-X-18
5	*Epeorus bispinosus*	China	Yunnan	Gaoligongshan National Nature Reserve	2020-VIII-20
6	*Epeorus rhithralis*	China	Yunnan	Jinping County	2020-X-20
7	*Epeorus* sp.2	China	Jiangxi	Jiulianshan National Nature Reserve	2020-IX-16
8	*Epeorus alexandri*	China	Inner Mongolia	Arxan	2014-VIII-25
9	*Epeorus* sp.3	China	Tibet	Lengjiaqu	2020-IX-16
10	*Epeorus herklotsi*	China	Zhejiang	Hangzhou City, Tianmu Mountain	2020-IV-05
11	*Epeorus dayongensis*	China	Guangxi	Huaping National Nature Reserve	2020-VIII-20
12	*Epeorus tuberculatus*	China	Zhejiang	Lishui City, Jingning County	2020-VIII-10
13	*Epeorus psi*	China	Yunan	Wenshan City	2020-X-17
14	*Epeorus* sp.4	China	Yunan	Nujiang River	2019-X-15
15	*Epeorus* sp.5	China	Yunan	Nujiang River	2020-IX-20
