# Supplementary material for: Comparative Mitogenome Analyses of Subgenera and Species Groups in Epeorus (Ephemeroptera: Heptageniidae)
Source: Insects. 2022 Jun 30;13(7):599. doi: 10.3390/insects13070599 (PMC9317806; doi:10.3390/insects13070599)
Supplement: Supplementary file 1 [file insects-13-00599-s001.zip › Table S3.pdf]

Table S3 Start and stop codons of 13 PCGs

Species \ Genes	ATP6	ATP8	COI	COII	COIII	CYTB	ND1	ND2	ND3	ND4	ND4L	ND5	ND6
Epeorus gibbus	ATA/TAA	ATG/TAA	ATT/TAA	ATG/T*	ATG/TAA	ATG/**T***	ATG/TAA	ATG/TAA	**ATC**/TAG	ATG/T*	ATG/TAA	**ATG**/T*	ATT/TAA
Epeorus unispinosus	ATA/TAA	ATG/TAA	ATT/**TAG**	ATG/T*	ATG/TAA	ATG/TAG	ATG/TAA	ATG/TAA	ATT/TAG	ATG/T*	ATG/TAA	GTG/T*	**TTG**/TAA
Epeorus sp.1	ATA/TAA	ATG/TAA	ATT/TAA	ATG/T*	ATG/TAA	ATG/TAG	ATG/TAA	ATG/TAA	**ATC**/TAG	ATG/T*	ATG/TAA	GTG/T*	**ATG**/TAA
Epeorus aculeatus	ATA/TAA	**GTG**/TAA	ATT/TAA	ATG/T*	ATG/TAA	ATG/TAG	ATG/TAA	ATG/TAA	ATT/TAG	ATG/T*	ATG/TAA	GTG/T*	ATT/TAA
Epeorus bispinosus	ATA/TAA	ATG/TAA	ATT/TAA	ATG/T*	ATG/TAA	ATG/TAG	ATG/TAA	ATG/TAA	ATT/TAG	ATG/T*	ATG/TAA	GTG/T*	ATT/TAA
Epeorus rhithralis	ATA/TAA	**GTG**/TAA	ATT/TAA	ATG/T*	ATG/TAA	ATG/TAG	ATG/TAA	ATG/TAA	ATT/TAG	ATG/T*	ATG/TAA	GTG/T*	ATT/TAA
Epeorus falcatus	ATA/TAA	ATG/TAA	ATT/TAA	ATG/T*	ATG/TAA	ATG/TAG	ATG/TAA	ATG/TAA	ATT/TAG	ATG/T*	ATG/TAA	GTG/T*	ATT/TAA
Epeorus melli	ATA/TAA	ATG/TAA	ATT/TAA	ATG/T*	ATG/TAA	ATG/TAG	ATG/TAA	ATG/TAA	**ATG**/TAG	ATG/T*	ATG/TAA	GTG/T*	ATT/TAA
Epeorus sp.2	ATA/TAA	ATG/TAA	ATT/TAA	ATG/T*	ATG/TAA	ATG/TAG	ATG/TAA	ATG/TAA	**ATG**/TAG	ATG/T*	ATG/TAA	GTG/T*	ATT/TAA
Epeorus carinatus	ATA/TAA	**GTG**/TAA	ATT/TAA	ATG/T*	ATG/TAA	ATG/TAG	ATG/TAA	ATG/TAA	**ATG**/TAG	ATG/T*	ATG/TAA	GTG/T*	ATT/TAA
Epeorus pellucidus	ATA/TAA	ATG/TAA	ATT/TAA	ATG/T*	ATG/TAA	ATG/TAG	ATG/TAA	ATG/TAA	ATT/TAG	ATG/T*	ATG/TAA	GTG/T*	ATT/TAA
Epeorus alexandri	ATA/TAA	ATG/TAA	ATT/TAA	ATG/T*	ATG/TAA	ATG/TAG	ATG/TAA	ATG/TAA	**ATC**/TAG	ATG/T*	ATG/TAA	GTG/T*	ATT/TAA
Epeorus montanus	ATA/TAA	ATG/TAA	ATT/TAA	ATG/T*	ATG/TAA	ATG/TAG	ATG/TAA	ATG/TAA	ATT/TAG	ATG/T*	ATG/TAA	GTG/T*	ATT/TAA
Epeorus sp.3	ATA/TAA	ATG/TAA	ATT/TAA	ATG/T*	ATG/TAA	ATG/TAG	ATG/TAA	ATG/TAA	ATT/TAG	ATG/T*	ATG/TAA	GTG/T*	ATT/TAA
Epeorus herklotsi	ATA/TAA	**GTG**/TAA	ATT/TAA	ATG/T*	ATG/TAA	ATG/TAG	ATG/TAA	ATG/TAA	ATT/TAG	ATG/T*	ATG/TAA	GTG/T*	ATT/TAA
Epeorus dayongensis 01	ATA/TAA	**GTG**/TAA	ATT/TAA	ATG/T*	ATG/TAA	ATG/TAG	ATG/TAA	ATG/TAA	**ATC**/TAG	ATG/T*	ATG/TAA	GTG/T*	ATT/TAA
Epeorus dayongensis 02	ATA/TAA	**GTG**/TAA	ATT/TAA	ATG/T*	ATG/TAA	ATG/TAG	ATG/TAA	ATG/TAA	**ATC**/TAG	ATG/T*	ATG/TAA	GTG/T*	ATT/TAA
Epeorus tuberculatus	ATA/TAA	**GTG**/TAA	ATT/TAA	ATG/T*	ATG/TAA	ATG/TAG	ATG/TAA	ATG/TAA	**ATC**/TAG	ATG/T*	ATG/TAA	GTG/T*	ATT/TAA
Epeorus psi	ATA/TAA	ATG/TAA	ATT/TAA	ATG/T*	ATG/TAA	ATG/TAG	ATG/TAA	**GTG**/TAA	ATT/TAG	ATG/T*	ATG/TAA	GTG/T*	ATT/TAA
Epeorus sp.4	ATA/TAA	ATG/TAA	ATT/TAA	ATG/T*	ATG/TAA	ATG/TAG	ATG/TAA	ATG/TAA	ATT/TAG	ATG/T*	ATG/TAA	GTG/T*	ATT/TAA
Epeorus sp.5	ATA/TAA	ATG/TAA	ATT/TAA	ATG/T*	ATG/TAA	ATG/TAG	ATG/TAA	ATG/TAA	ATT/TAG	ATG/T*	ATG/TAA	GTG/T*	ATT/TAA
