# Supplementary material for: Comparative Mitogenome Analyses of Subgenera and Species Groups in Epeorus (Ephemeroptera: Heptageniidae)
Source: Insects. 2022 Jun 30;13(7):599. doi: 10.3390/insects13070599 (PMC9317806; doi:10.3390/insects13070599)
Supplement: Supplementary file 1 [file insects-13-00599-s001.zip › Table S5.pdf]

Table S5 Primers for mitogenomes of *Epeorus* used in this study

Fragment	Primer	Primer sequences	Reference
Part of COI	LCO1490	5'-GGTCAACAAATCATAAAGATATTGG-3'	[29]
	HCO2198	5'-TAAACTTCAGGGTGACCAAAAAATCA-3'	
Part of rrnL	16Sa	5'-GCCTGTTTATCAAAAACAT-3'	[30]
	16Sb	5'-CTCCGGTTTGAACTCAGATCA-3'	
Fragment of block (I-M-Q-M/NCR-ND2)	1-ND2-F	5'-GATGCCTGACAAAAGGGCTAC-3'	This work
	1-ND2-R	5'-GGCTCAAAGGTAAGAGGCGA-3'	
	2-ND2-F	5'-TGCCTGACAAAAGGGCTACTT-3'	
	2-ND2-R	5'-CAAAGGGTTCAGGGGCTCAT-3'	
	3-ND2-F	5'-CAAAGGGTTCAGGGGCTCAT-3'	
	3-ND2-R	5'-TGGTTTGTGTGGTCAGGCTTA-3'	
	4-ND2-F	5'-TGAAATGCCTGATAAAAGGACTACT-3'	
	4-ND2-R	5'-CCCAGTCAAGCTCCCATTCA-3'	
	5-ND2-F	5'-ATGAAATGCCTGATAAAAGGACTAC-3'	
	5-ND2-R	5'-TATGGCAATGCTAAGGGGCA-3'	
	6-ND2-F	5'-ATGAAATGCCTGATAAAAGGACTAC-3'	
	6-ND2-R	5'-AAAAGGACGGCGGAAGCTAA-3'	
	7-ND2-F	5'-TGAAATGCCTGATAAAAGGGTTAC-3'	
	7-ND2-R	5'-AGCAAGGGCCTGTGTAAGAA-3'	
	8-ND2-F	5'-TGCCTGATTAAAGGGCTACTTTG-3'	
	8-ND2-R	5'-GGTAGTGCGCCCGTAAATCT-3'	
	9-ND2-F	5'-TAGATCATGTAGGCCACCCT-3'	
	9-ND2-R	5'-CAAGCCAAGCACCCATTCAT-3'	
	10-ND2-F	5'-ATCATGTAGGGTTCCTACTTTCATT-3'	
	10-ND2-R	5'-AGAAGTGAAAAGGGGCTGCTC-3'	
	11-ND2-F	5'-TAAGGAAAGATAAGCTAACTGCAAG-3'	
	11-ND2-R	5'-CCTAGCCATGCTCCTAACCAA-3'	
	12-ND2-F	5'-TCATGTAGGGTTCCTACTTTCATT-3'	
	12-ND2-R	5'-AAGCTGCCTCGGTAGCAAAT-3'	
	13-ND2-R	5'-TGAAATGCCTGATAAAAGGACTACT-3'	
	13-ND2-R	5'-AGTCAAGCTCCCATTCAGGA-3'	
	14-ND2-R	5'-CATGTAGGCCTCCCTACTTTCAT-3'	
	14-ND2-R	5'-TCCAAACCTAACCAAGCACCT-3'	
	15-ND2-R	5'-ATGAAATGCCTGATAAAAGGGCT-3'	
	15-ND2-R	5'-GCGAGAGCTTGGGTGAGAAA-3'	
