# Supplementary material for: Comparative Mitogenome Analyses of Subgenera and Species Groups in Epeorus (Ephemeroptera: Heptageniidae)
Source: Insects. 2022 Jun 30;13(7):599. doi: 10.3390/insects13070599 (PMC9317806; doi:10.3390/insects13070599)
Supplement: Supplementary file 1 [file insects-13-00599-s001.zip › Table S6.pdf]

Table S6 Partition schemes and best-fitting models selected in PCG dataset.

	**Nucleotide sequence alignment**	
**Partition**	**Partition name**	**Best model**
1	COI_codon1	SYM+G
2	COI_codon2, CO3_codon2, COII_codon2, ATP6_codon2, Cytb_codon2	GTR+I
3	COI_codon3, ATP6_codon3, ATP8_codon3, COII_codon3	HKY+I+G
4	ND4L_codon1, ND1_codon1, ND4_codon1, ND5_codon1	GTR+I+G
5	ND4L_codon2, ND1_codon2, ND4_codon2, ND3_codon2, ND5_codon2	GTR+I+G
6	ND1_codon3, ND4L_codon3, ND5_codon3, ND4_codon3	GTR+I+G
7	ATP6_codon1, CO3_codon1, COII_codon1, Cytb_codon1	SYM+G
8	ND3_codon1, ATP8_codon1, ND6_codon1, ND2_codon1	HKY+I+G
9	ND6_codon2, ND2_codon2, ATP8_codon2	GTR+I+G
10	CO3_codon3, ND2_codon3, Cytb_codon3, ND3_codon3, ND6_codon3	GTR+I+G
